# Supplementary material for: Anti-Idiotypic VHHs and VHH-CAR-T Cells to Tackle Multiple Myeloma: Different Applications Call for Different Antigen-Binding Moieties
Source: Int J Mol Sci. 2024 May 22;25(11):5634. doi: 10.3390/ijms25115634 (PMC11171536; doi:10.3390/ijms25115634)
Supplement: Supplementary file 1 [file ijms-25-05634-s001.zip › ijms-2957086-supplementary.pdf]

Supplementary Figure S1: DNA sequence of the of the used CAR construct. The used CAR construct includes an EF1*α* core promotor (highlighted in light green), a Kozak sequence (highlighted in black), an Igκ leader sequence (highlighted in pink), a VHH multiple cloning site (highlighted in cyan), a murine CD8*α*-derived hinge region (highlighted in khaki), a murine CD8*α*-derived transmembrane region (highlighted in grey), a murine 4-1BB co-stimulatory region (highlighted in yellow), and a murine CD3ζ T-cell activation domain (highlighted in dark green). EF1*α* = eukaryotic translation elongation factor 1*α*; mu = murine.

GGGCAGAGCGCACATCGCCCACAGTCCCCGAGAAGTTGGGGGGAGGGGTCGGCAATTGATCCGGTGCC
TAGAGAAGGTGGCGCGGGGTAAACTGGGAAAGTGATGTCGTGTACTGGCTCCGCCTTTTTCCCGAGGG
TGGGGGAGAACCGTATATAAGTGCAGTAGTCGCCGTGAACGTTCTTTTTCGCAACGGGTTTGCCGCCA
GAACACAGGACCGGTTCTAGAGGGATCCCACC**ATGGAGACAGACACACTCCTGCTATGGGTACTGCTG**
CTCTGGGTTCCAGGTTCCACTGGTGATCAGGTGCAGCTGCAGGAGTCTAGAGGGGACCCAGGTCACCG
TCTCCTCAACCACCACAAAACCTGTTCTGCGAACCCCTAGCCCCGTGCACCCCACCGGGACCTCCCAA
CCTCAGAGGCCAGAAGATTGCCGGCCACGCGGGTCCGTTAAAGGGACTGGGTTGGATTTCGCTTGCGA
TATCTATATATGGGCACCCTTGGCCGGGATCTGTGTTGCTCTTCTTCTTTCCCTCATTATAACACTCA
TATGTTACCAT**AGTGTTCTGAAGTGGATTAGAAAGAAGTTTCCACACATTTTTAAGCAGCCTTTCAAA**
AAGACCACCGGTGCAGCCCAAGAAGAGGACGCTTGCTCATGTAGGTGTCCTCAGGAGGAAGAAGGGGG
CGGGGGTGGCTACGAACTTCGCGCCAAGTTTAGCCGCTCCGCTGAAACCGCCGCCAATCTTCAAGATC
CAAATCAGCTCTATAACGAGCTGAACCTGGGTAGACGGGAAGAATATGACGTATTGGAAAAGAAGCGC
GCGCGAGACCCTGAAATGGGGGGCAAACAGCAACGAAGACGAAACCCCCAGGAGGGAGTGTATAATGC
CCTGCAAAAGGACAAGATGGCCGAGGCTTACAGCGAGATTGGAACCAAGGGAGAAAGAAGGCGAGGTA
AGGGGCACGATGGTCTGTATCAAGGACTTTCCACTGCCACCAAGGATACTTATGACGCCCTCCACATG
CAAACCTTGGCCCCTAGA

EF1α core promoter - Kozak sequence - Igκ leader sequence - Multiple cloning site for VHH sequences - muCD8α hinge region - muCD8α transmembrane region - - mu4-1BB co-stimulatory domain - muCD3ζ T-cell activation domain
